# Frailty assessment in critically ill older adults: a narrative review

**DOI:** 10.1186/s13613-024-01315-0

**Published:** 2024-06-18

**Authors:** L. Moïsi, J.-C. Mino, B. Guidet, H. Vallet

**Affiliations:** 1 Department of Geriatrics, Hopital Saint-Antoine, Assistance Publique Hôpitaux de Paris (AP-HP), Sorbonne Université, 75012 Paris, France; 2 UVSQ, INSERM, Centre de Recherche en Epidémiologie Et Santé Des Populations, UMR 1018, Université Paris-Saclay, Université Paris-Sud, Villejuif, France; 3 Département d’éthique, Faculté de Médecine, Sorbonne Université, Paris, France; 4 Service de Réanimation Médicale, Hopital Saint-Antoine, Assistance Publique Hôpitaux de Paris (AP-HP), 184 Rue du Faubourg Saint-Antoine, 75012 Paris, France; 5 INSERM, UMRS 1136, Institute Pierre Louis d’Épidémiologie Et de Santé Publique, 75013 Paris, France; 6 UMRS 1135, Centre d’immunologie Et de Maladies Infectieuses (CIMI), Institut National de La Santé Et de La Recherche Médicale (INSERM), Paris, France; 7 Service de Gériatrie Aigue, Hopital St Antoine, 184 rue du Fbg St Antoine, 75012 Paris, France

**Keywords:** Critical care, Older adults, Aged, 80 and over, Frailty, Frailty scales

## Abstract

Frailty, a condition that was first defined 20 years ago, is now assessed via multiple different tools. The Frailty Phenotype was initially used to identify a population of “pre-frail” and “frail” older adults, so as to prevent falls, loss of mobility, and hospitalizations. A different definition of frailty, via the Clinical Frailty Scale, is now actively used in critical care situations to evaluate over 65 year-old patients, whether it be for Intensive Care Unit (ICU) admissions, limitation of life-sustaining treatments or prognostication. Confusion remains when mentioning “frailty” in older adults, as to which tools are used, and what the impact or the bias of using these tools might be. In addition, it is essential to clarify which tools are appropriate in medical emergencies. In this review, we clarify various concepts and differences between frailty, functional autonomy and comorbidities; then focus on the current use of frailty scales in critically ill older adults. Finally, we discuss the benefits and risks of using standardized scales to describe patients, and suggest ways to maintain a complex, three-dimensional, patient evaluation, despite time constraints. Frailty in the ICU is common, involving around 40% of patients over 75. The most commonly used scale is the Clinical Frailty Scale (CFS), a rapid substitute for Comprehensive Geriatric Assessment (CGA). Significant associations exist between the CFS-scale and both short and long-term mortality, as well as long-term outcomes, such as loss of functional ability and being discharged home. The CFS became a mainstream tool newly used for triage during the Covid-19 pandemic, in response to the pressure on healthcare systems. It was found to be significantly associated with in-hospital mortality. The improper use of scales may lead to hastened decision-making, especially when there are strains on healthcare resources or time-constraints. Being aware of theses biases is essential to facilitate older adults’ access to equitable decision-making regarding critical care. The aim is to help counteract assessments which may be abridged by time and organisational constraints.

## Introduction

The population of older adults is growing, notably in the European Union (EU). Indeed, the proportion of over 65 year-olds grew by 3% between 2012 and 2022 [1]. Correspondingly, epidemiological studies point towards an increase in proportion of over 80 year-olds in Intensive Care Units (ICUs) [2, 3]. In this population, hospitalization must focus on functional outcomes (preserving one’s functional ability, health-related quality of life, being discharged home after a hospital stay) as opposed to only focusing on mortality. For some years, the notion of frailty and the various scores used to assess it have been used as tools to help in the decision to admit older adults to the ICU. Though determining which patients might most benefit from intensive care is essential, there are no guidelines or consensus regarding the admission of critically ill older adults to intensive care [4].

**Fig. 1:**
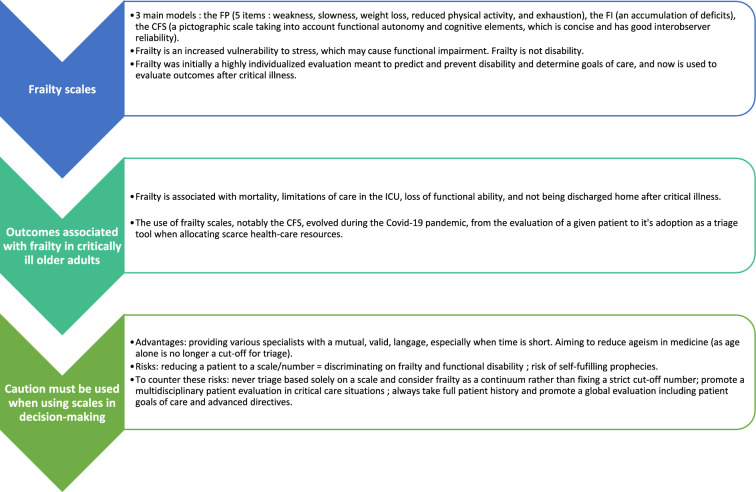
Key concepts discussed in the article. *FP* Frailty phenotype, *FI* Frailty Index, *CFS* Clinical frailty scale, *ICU* Intensive care unit

Thus, we aimed to describe different frailty assessment tools used in over 75 year-old critically ill patients, as well as distinguish them from functional autonomy and comorbidity scales; review the current existing literature regarding frailty and critically ill older adults; discuss limits to the use of scales as a tool, and which scales might be the most appropriate in emergency situations. The main concepts discussed in the article are summarized in Fig. 1.

## A history of frailty, functional autonomy, and comorbidity scales

The concepts of functional autonomy, frailty, and comorbidities were created starting in the 1960s and 1970s, following the development of geriatrics. Geriatrics, a newer specialty, emerged in the UK in the 1930s, and then in the USA in the 1940s. As geriatrics developed, so did the concept of the comprehensive geriatric assessment (CGA) [5]. CGA is a method providing thorough patient appraisal, using a number of scales and scores, thus including multiple aspects relevant to care of older adults (functional autonomy, cognition, mental health status, nutrition). Understanding and differentiating the key concepts of comorbidity, functional autonomy and frailty is crucial, both to CGA, and to discussing appropriateness of care in case of acute illness.

### Comorbidities

The concept of comorbidity was first mentioned by the epidemiologist Feinstein in 1970. He noted that comorbidities are a main confounding factor for aetiology and prognosis studies, as they can majorly influence healthcare outcomes of an index disease [6]. The most common comorbidities are hypertension, diabetes, chronic obstructive pulmonary disease, cardiac failure, cancer and cognitive impairment [7]. In a critical review, De Groot, et al. identify 13 methods to assess comorbidities [8], of which two are the most relevant for geriatric practice. The Charlson Comorbidity Index (CCI) is the most extensively studied for predicting mortality. Developed in late 1980s, it’s described as a “weighted index, taking into account the number and severity of comorbid diseases”, and includes 19 different comorbidities [9]. A combined score of comorbidities and age predict 10 year mortality, a higher score being associated with a significant decrease in survival. An age-adjusted CCI was validated in 1994 [10]. The Cumulative Illness Rating Scale (CIRS), first developed in 1968, addresses 13 different bodily systems, without using specific diagnoses, scored between 0 and 4. It was then modified in 1992, resulting in today’s CIRS-G (for geriatric) scale, which was also validated as an independent predictor of mortality at 18 months [11]. The Elixhauser comorbidity index, which is broader as it includes 31 comorbidities, has been proven to be useful in predicting 30 day-mortality in over 75 year-old patients in acute care, though it does not clearly include dementia [12, 13].

Comorbidities, evaluated using the CCI, are associated with poor outcomes in ICU patients, including in-hospital [14], 30 day [15], 3 month [16], and 1 year mortality [15]. However, as comorbidity scales are time-consuming, they are not suitable for daily use, especially in emergency situations. Thus, functional autonomy and frailty scales are more commonly used in critically ill patients.

### Functional autonomy scales

The main functional autonomy (FA) scales used in geriatrics are Katz’s Activities of Daily Living (ADL) scale, and Lawton and Brody’s Instrumental Activities of Daily Living (IADL) scale [17]. The ADL scale was first developed in 1968, to assess the functional autonomy of stroke and hip surgery patients after rehabilitation or in long-term care, so as to evaluate which type of assistance they required upon discharge [18]. The Barthel Index, created in 1965 to evaluate a patient after acute illness undergoing rehabilitation (similar to the ADL scale but more detailed), is less commonly used among geriatricians [19]. The Performance Status (PS), created in 1982 [20], is also widely used, mainly in patients with cancer. However, as the PS is a lot less precise, it is poorly suited to evaluate older adults.

Gradually, these scales have evolved to be used to evaluate patient prognosis in critically ill situations, including upon admission to critical care units. In recent years, baseline functional status was found to be significantly associated with in-hospital mortality [21], ICU and 1 month mortality [22], and 6 to 12 month mortality [23, 24] after a critical illness requiring an ICU stay. In addition, there is significant loss of FA 1 year after critical illness resulting in ICU admission. Moreover, a lower baseline functional autonomy was found to be associated with a higher risk of deterioration [25].

### Frailty scales

Frailty is theoretically defined as “a clinically recognizable state of increased vulnerability, resulting from age associated decline in reserve and function across multiple physiological systems” [26]. In such, in frail patients, the ability to cope with both every day and acute stressors is compromised. The notion of frailty has evolved over the years. The frailty phenotype (FP), a predictive model created in 2001 by Fried et al. [29], was designed to identify frail patients in a community setting, by at least three of five following criteria: fatigue, weight loss, reduction of gait speed, muscle weakness, and lack of exercise [27]. Those with none of the above characteristics were considered robust, pre-frail if they had one or two of the criteria, and frail if they had more than three criteria. In a large cohort of over 65 year-olds, frailty was associated with a higher risk of falls, worsening mobility, hospitalization. It was also strongly associated with long-term mortality, the risk of dying ranging from 1.5 to 2.5 times higher at 3 and 7 years as compared to robust patients. Long-term mortality was also higher for frail patients than pre-frail patients.

The frailty index (FI) was developed the same year by Rockwood and his team [28], based on the Canadian Health Studies in Aging (CHSA) cohort. Their intent was to develop a tool to reflect a patient’s health status and that of a group of individuals, thus providing an estimation of aging and mortality. The FI is based on 92 types of health deficits, accumulated during the course of life: symptoms (low mood, changes in sleep), signs (tremor, decreased peripheral pulses), abnormal laboratory values (urea, creatinine), disease classifications (diabetes mellitus, Parkinson’s disease), and disabilities (dependence in bathing or dressing, signs, functional disabilities, laboratory anomalies). These deficits range from discomfort (i.e., constipation, skin problems), to those associated with a higher risk of death (i.e., cancer). The FI, by taking into account a full range of deficits, is meant to give an estimation of “biological age” vs “chronological age”, thus the risk of mortality. Though very complete, its length renders it inconvenient for emergency use.

In 2005, Rockwood et al. developed a 7-point Clinical Frailty Scale (CFS), based on a multidimensional approach including fitness, functional autonomy and comorbidities. Each number/category of the scale describes a different stage of this combined approach, based on a healthcare professional’s clinical judgment. Each 1-category increment increased the risk of death at 5 years by 21%, and the risk of entry to an institution by 23% [29]. The scale then evolved in 2007 to the 9 point-CFS, a visual scale classifying patients from fit (CFS 1–3), to vulnerable (CFS 4), to frail and more (CFS 5–9). Adding “very severely frail” and “terminal illness” in 2007 to the classification enabled distinguishing severely frail patients (CFS 7) from bedridden (CFS 8) and terminal patients (CFS 9). The CFS was then revised again in 2020 in the context of the Covid-19 pandemic, modifying categories 2 (from “well” to “fit”) and 4 (from “vulnerable” to “living with very mild frailty”) and specifying the measure of frailty in patients with cognitive impairment [30].

Multiple tools are accessible to help clinicians train with the CFS [31, 32]. Studies have shown its reliability between evaluators and across specialities and professions [33, 34], and in different languages [35].

Other frailty scales, based on patient records and administrative coding, include the Hospital Frailty Risk Score (HFRS), validated in 2018 by Gilbert et al. The HFRS evaluates frailty based on patient records, and was shown to predict 30 day-mortality, length of hospital stay and 30 day readmission [36]. However, access to computerized records is an ongoing challenge in many countries. In addition, because the HFRS is based on administrative records, information may lack when patients have never been admitted to the hospital, and the score may be subject to measurement error and not reflect disease severity. One study comparing the HFRS and the CFS found a weak correlation between the two, and that the CFS was a better predictor of 1 year mortality [37]. It seems that these means of evaluating frailty, derived from administrative codes, may be in fact closer to an evaluation of comorbidity than to a more conventional geriatric frailty assessment and not suitable for critical care situations [38].

**Fig. 2:**
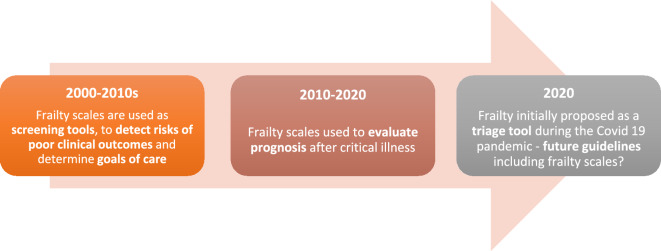
Evolution of the use of frailty scales over time

Because the concept of frailty has evolved over the years, geriatricians themselves use different tools to evaluate frailty, and standardization is needed [39]. In a scoping review of frailty assessment in the acute care setting, 25% of the articles reviewed used non-frailty tools (such as the Short Physical Performance Battery, grip strength, or the Barthel Index) to evaluate frailty [40]. Indeed, frailty is a broad concept, the meaning currently ranging from “physical weakness, sarcopenia” to the preventive, biological, psychological and social meaning of frailty, mostly used in ambulatory patients [41]. Thus, it is particularly important when discussing frailty to specify which definition and which frailty scale is one using, as well as one’s purpose**. **Figure 2 depicts the evolution in the use of frailty scales since the year 2000. Table 1 summarizes the different methods to evaluate frailty, their advantages and drawbacks, and relation to patient outcomes.

**Table 1:** Different frailty assessment models, advantages/drawbacks and relation to outcomes

Frailty scale	Components	Frailty classification	Advantages/drawbacks	Relation to outcomes
Frailty phenotype [25]	Five items: weight loss, low physical activity, exhaustion, slowness, weakness	Robust: 0 items Pre-frailty: 1–2 items; Frailty: ≥ 3 items	• A comprehensive model, part of a CGA • Useful to evaluate patient trajectories, loss of function and disability in non-life-threatening situations • Information may be hard to obtain in an emergency situation	Evaluating outcomes in over 75 year-olds after critical illness would require further study
Frailty index [26]	30 to 92 accumulated health deficits Scores range from 0 (no deficits) to 1 (all deficits are present, though the maximum found is 0.7)	Continuous score Suggested cut-off score for frailty > 0·25	• Comprehensive model which gives an estimation of biological age vs chronological age • Can be applied to most health data sets • Time-consuming, so inconvenient for emergency use	Predicts mortality, disability, and cognitive decline better than chronological age Is associated with long-term mortality, place of residence and physical recovery after critical illness
Clinical frailty scale [20, 27, 28, 48, 52–54, 56]	Visual chart with a description of each picture 9 stages, ranging from 1 (very fit) to 9 (terminally ill)	CFS ≥ 5 defines frail	• Visual tool which easily conveys information • Good interrater reliability • May entail an evaluation based solely on the visual scale • At risk of use for inappropriately conducted triage	Outcomes after critical illness have been frequently studied CFS is a strong predictor of mortality, loss of functional ability, and not being discharged home
Scales based on administrative (computarized) records: the hospital frailty risk scale [34, 36]	109 summed items from ICD-10 frailty-relevant codes from administrative hospital data	HFRS: Low risk: score < 5 intermediate risk: score 5–15; high risk: score > 15	• Can be calculated automatically upon patient admission and discharge • Frailty assessment subjected to lack of data and measurement bias • May not reflect disease severity • Higher correlation with comorbidities than with a frailty evaluation	Inadequate predictors of long-term survival after critical illness

*CFS* Clinical frailty scale, *CGA* Comprehensive geriatric assessment, *HFRS* Hospital frailty risk scale, *ICD* International classification of diseases

Frailty has been found to predict patient outcomes in different countries and settings. A recent review, including 26 studies, found significant association between the CFS in over 65 years-olds, all-cause mortality, and functional decline after admission to acute hospital settings [42]. After an acute illness, the CFS was found to be an independent predictor both of 30 day and 6 month-mortality. This remained true even when illness severity was low [43]. One study found a strong association between the CFS and being discharged home after being admitted for Covid-19 (OR = 13.44, IC 95% (3.98–45.37), p < 0.001) [44].

In recent years, frailty has increasingly been studied in the context of critical care. In 2014, two studies, enrolling over 50 year-old and 65 year-old critically ill patients, found frailty to be an independent predictor of ICU mortality, in-hospital mortality, 1 year mortality, and functional disability [45, 46]. Confirming the importance of frailty, a study including over 50 year-olds found frailty to be significantly associated with long-term outcomes defining health related quality of life, such as loss of functional ability, higher pain and discomfort, and higher anxiety and depression [47]. This spiked the intensive care community’s interest in frailty, which up until that point was mainly studied by geriatricians. Indeed, the number of publications studying frailty in critically ill older adults [Pubmed search for frailty AND (“critically ill” OR “critically illness” OR “intensive care” OR “critical care” OR “icu”) AND (“elderly” OR “old” OR “older” OR “ > 75”)] evolved from 6 in 2008 to 1189 in 2024.

### Overlap between comorbidities, functional autonomy and frailty

Caution is needed when discussing functional autonomy and frailty. Though there is overlap between comorbidities, frailty and ADL dysfunction, they remain separate entities. Comorbidities are risk factors for frailty, and frailty is a risk factor for disability. Data from the VIP2 study shows that there is important overlap between the ADL scale and frailty, 43% of patients presenting overlapping syndromes [48]. In addition, the CFS and the ADL scale may be tricky to use for patients of intermediate frailty or functional autonomy. For instance, the difference between a “CFS-4” and “CFS-5” patient may be difficult to determine. One could argue that living with very mild frailty and mild frailty is a continuum. Indeed, a recent meta-analysis found that frailty defined by the CFS is a continuum in regards to ICU mortality, confirming that all CFS categories must be considered individually, as opposed to the suggested initial pooling of categories (CFS 1–3, CFS 4, CFS 5–9) [49]. In addition, the state of a patient may vary from one to the other and back over some months.

Rockwood suggests that “if a patient fits two categories equally well, in routine care it is best to score the scale at the higher or more dependent level” [30]. Thus, it is particularly important to clarify the role of frailty itself, which has extensively been studied in the context of critically ill over 75 year-olds.

## Frailty in critically ill over 75 year-olds

### Definition and prevalence of “frailty” in critically ill over 75 year-olds

A majority of studies use the CFS, based on patient status before admission, to evaluate frailty [50]. Frailty is routinely defined by a CFS of more than 4 or 5. Hope et al. used a self-defined health category, ranging from “feeling robust, frail, having chronic organ failure or cancer” [51] to define frailty. Heyland et al. used the Frailty Index established via a comprehensive geriatric assessment (frailty ranging from by mild (0–0,2), to moderate (0,2–0,4), to severe (> 0.4) [52]. The VIP1 and 2 studies found frailty to be common in ICU patients. Indeed, 40% of patients had a CFS of 5 or more [22, 53].

Most studies examined ICU patient cohorts, either general or medical ICUs. Only one study was set in the emergency room, integrating critically ill patients presenting to the ER [54]. We found no studies taking place in acute care (geriatric or other), defining older adults as “critically ill”. Main frailty scales used in critically ill older adults and associated outcomes are presented in Table 2.

**Table 2:** Main studies using frailty scales in critically ill over 75 year-olds and associated outcomes

Authors/year country	Frailty tool used	Study design	Age category, number of patients	Population (ICU, acute care med, ER.)	Outcome measure
Bruno et al. 2020 Germany	CFS ≥ 5 (baseline assessment at home before ICU)	Prospective cohort study (data merged from the VIP1 and 2 cohorts)	Age ≥ 80 yrs, N = 415	Admission to ICU	30 day mortality prediction: frailty per CFS adjusted OR = 1.23 (1.04–1.46), p = 0.02 Treatment withdrawal/withheld: CFS > 4, 62.7% vs 47.4%, p = 0.009
Darvall et al. 2019 Australia and New Zealand	CFS ≥ 5 (preadmission at home 2 months before hospital admission)	Retrospective cohort study	Age ≥ 80 yrs, N = 15613	Admission to ICU	Frailty was significantly associated with in-hospital mortality adjusted OR, 1.87; 95% CI, (1.65–2.11) Discharge to nursing home or chronic care: adjusted OR = 1.61 (1.34–1.95) < 0.0010.82 (0.80–0.83)
De Geer et al. 2022 Sweden	CFS ≥ 5 defines frailty, assessed after admission based on prior patient status	Prospective observational study	Subgroups of patients ages 18-49, 50-64, 65-79, ≥ 80 yrs (N = 77/871)	All patients admitted to a mixed tertiary ICU	6 month mortality rates Unadjusted analysis = HR frail vs non-frail = 2.4 (1.1–5), p = 0.02 for ≥ 80 year olds HR not significant in the multivariate analysis
De Lange et al. 2019 Europe	CFS (at ICU admission)	Prospective cohort study	Age > 80 yrs, N = 3730	ICU	CFS is one of several independent predictors of 30 day mortality OR = 1.19 (1.14-1.25)
Fisher et al. 2015 Australia	CFS (0–4 non frail, 5-6 mild frailty, ≥ 7 severely frail)	Prospective cohort study	2 age groups, > 65 yrs and > 85 yrs, N = 348	ICU admission (all except palliative care and organ donation)	No association between frailty and ICU mortality, hospital mortality or discharge location. Weak correlation with increased hospital LOS
Flaatten et al. 2017 Europe	CFS ≥ 5 (directly before H admission), CFS 4 prefrail, CFS 1-3 not frail	Prospective cohort study (VIP 1)	Age ≥ 80 yrs, N = 5021	ICU	HR for survival analysis (censored at 30 days): Vulnerable (4) vs fit (1–3) 1.19 (1.03–1.38); Frail (5–9) vs fit (1–3): 1.54 (1.38–1.73) < 0.001 Stronger association in acute vs elective admissions
Fronczek et al. 2018 Poland	CFS ≥ 5 (timing unspecified)	Subgroup of a prospective cohort study (VIP1)	Age ≥ 80 yrs, N = 272	ICU admission	Adjusted ICU mortality when frail = 2.25 (1.26–4.01) (p = 0.006)
Guidet et al. 2020 Europe	CFS ≥ 5, CFS 4 “prefrailty”	Prospective cohort study (VIP2)	Age ≥ 80 yrs, N = 3920	ICU admission	30 day mortality prediction CFS (per point): 1.1 (1.05–1.15, p < 0.001). Analysis of treatment limitation CFS (per point): 1.08 (IC 95% 1.05-1.12), p < 0.0001
Haas et al. 2021 Europe	CFS > 4 defines frailty	Prospective cohort study	Age ≥ 80 yrs, SOFA ≥ 2 with sepsis N = 532	Acutely admitted ICU patients	6 month-mortality for frail vs fit patients: HR = 1.34 (95% CI 1.18–1.51), p < 0.0001
Heyland et al. 2015 Canada	FI based on CGA (2 weeks before hospitalization) Mild 0–0,2; moderate 0,2–0,4; severe > 0.4	Prospective observational cohort study	Age ≥ 80 yrs, N = 610	ICU admission at least 24 h	1 yr survival rate = 50% 34% discharged home at 12 months, correlated to the FI (p = 0.02) Physical recovery defined by being alive + combination of baseline status + SF36 Recovery = 26% at 12 months, significantly associated with the CCI (p = 0.03) et the FI (p < 0.0001)
Heyland et al. 2016 Canada	CFS	Prospective longitudinal cohort study	Age ≥ 80 yrs, N = 527	ICU admission, excluded elective surgery admission Medical or surgical emergency care	CFS was an independent predictor of recovery from ICU illness, defined by a Palliative Performance Status > 60 (PPS = 60: reduced ambulation, occasional assistance) OR per 2 pts CFS = 0.42 (IC 0.25–0.71), p = 0.001
Hope et al. 2015 USA	4 self-defined health categories: robust, chronic organ failure, cancer, frailty During the year before ICU admission	Retrospective cohort study	Age ≥ 66 yrs, age groups 66–70, 70–79, and ≥ 80 yrs; N = 47 427	ICU	Patients with pre-ICU frailty: higher hospital and 3 yr mortality (in frailty vs robust alone and frailty + cancer; frailty + chronic organ failure)
Huh et al. 2022 Japan	CFS ≥ 5 defines frailty Compared CFS 1–4 patients to CFS5, 6, 7–9 patients CFS corresponds to state before admission	Retrospective observational study	Age ≥ 75 yrs, N = 544	Patients presenting to the ER subsequently admitted to the ICU Out of hospital cardiac arrests and transfers excluded	Adjusted risk ratio of 30 day mortality: RR = 1.45 (0.87–2.41) for CFS 5; RR = 1.85 (1.13 to 3.03) for CFS 6; RR = 2.44 (1.50 to 3.96) for CFS 7–9
Ibarz et al. 2020 Europe	CFS “fit CFS ≤ 3”, “vulnerable (CFS = 4)”, “frail” CFS ≥ 5 before H admission	Prospective cohort study (post-hoc analysis of the VIP1 study)	Age ≥ 80 yrs, N = 3869	Acute ICU admission	Survival rate at 30 days, Frail vs Fit:1.47 (1.07 2.02), p = 0.0182
Kasapoglu et al. 2021 Turkey	CFS ≥ 5 defines frailty, based on patient status before admission	Retrospective cohort study	Age ≥ 80 yrs, N = 133	ICU stay ≥ 24 h	CFS predicted short-term mortality (CFS ≥ 5 mean survival time (days): 20.31 ± 1.25 (IC 17,86–22,79) vs 29.49 ± 0.50 (IC 28.5–30.48), p < 0.0001
Muessig et al. 2018 Germany	CFS before H admission	Prospective cohort study (subgroup of the VIP1 study)	Age ≥ 80 yrs, N = 308	ICU admission	30 day mortality Frailty per-point increase adjusted OR = 1.437 (IC 1.052-1.964), p = 0.023
Orsini et al. 2016 USA	Simplified CFS CFS ≥ 5 defines frailty	Prospective cohort study	Age ≥ 80 yrs, N = 52	ICU admission	Mean CFS similar in survivors and non survivors
Pasin et al. 2020 Italy	CFS ≥ 5 defines frailty	Unmatched case-control study	Age ≥ 80 yrs, N = 302	ICU admission for medical reasons	CFS was associated with 1 year mortality (mortality 84% vs 65%, p < 0.001) but not 30 day mortality
Wernly et al. 2020 Europe	CFS Timepoint unspecified	Secondary analysis of 2 prospective multicenter cohort studies (VIP1 et 2)	Age ≥ 80 yrs, N = 7555	ICU admission	Frailty was independently associated with 30 day mortality in male patients (adjusted OR 1.13 (1.09-1.18), p < 0.001 and female patients (AOR 1.16 (1.10–1.21), p < 0.001

*CCI* Charlson comorbidity index, *CFS* Clinical frailty scale, *FI* Frailty index, *HR* Hazard ratio, *ICU* Intensive care unit, *LOS* Length of stay,*OR* Odds ratio, *RR* Relative risk, *SF36* Short form 36 score, *VIP* Very old intensive care patient

### Outcomes associated with frailty

Most studies found that frailty was an independent predictor of short and long-term mortality after a critical illness. Darvall et al., in a population-based cohort study in Australia and New Zealand, found significant association between frailty and in-hospital mortality (adjusted OR, 1.87; 95% CI, (1.65–2.11) [55]. Fronczek et al., in a Polish subgroup of the VIP1 study, found that ICU-mortality was 2.25 times higher in frail patients [56]. Frailty was also significantly associated with short-term [57] and 1 month-mortality [22, 54, 58]. Flaatten et al., in the VIP1 study, found that this was true for both vulnerable patients and frail patients [53]. They also found a higher rate of treatment limitation as the CFS increased [HR per one point CFS-increase = 1.11 (95% CI 1.08–1.15)]. This confirms that the higher mortality rate in the ICU in frail older adults may be related to a higher rate of limitations of life-sustaining treatments in this population [59]. This points out that self-fulfilling prophecies may play a part in the mortality rate. Frailty was also associated with longer length of stay (LOS), both in the ICU and in the hospital [55, 60].

Similarly, Haas et al. found a significant association between frailty and mortality at 6 months for septic frail vs septic fit patients [61]. Pasin et al. found that mortality was also higher in frail patients 1 year after ICU stay [62] (84 vs 65% in fit patients, p < 0.001). Hope et al., in their retrospective cohort using Medicaid records, established that this was still true after 3 years (adjusted HR for pre-ICU frail patients vs non-frail patients ranging from 1.54 (95% CI 1.45–1.64) to 1.84 (95% CI 1.70–1.99) [51].

Several studies evaluated the loss of functional ability after critical illness. Heyland et al. found that a higher FI was significantly associated with not recovering from baseline status 1 year after critical illness [52]. “Physical recovery” was defined by being alive and combining baseline status and Short-Form 36 (SF36) scores. Recovery was 26% at 12 months, and inversely associated with the Frailty Index. In another study, Heyland found the CFS to be significantly associated with recovery at 1 year (recovery defined by a Palliative Performance Status of ≥ 60, equivalent to reduced ambulation, being unable to do house work, needing occasional assistance with self-care, having normal or reduced food/drink intake, and having full conscience or being confused) [63].

Furthermore, frailty is associated with one’s place of residence after critical illness. Heyland et al. found frailty to be significantly associated with home discharge at 1 year (fit patients (FI < 0.2), 39% were discharged home, vs 26% for frail patients (FI > 0.4), p = 0.02) [52]. Darvall et al. encountered a strong association between being newly discharged to a nursing home or chronic care 1 year after critical illness (OR for frail vs non-frail patients = 1.61 (1.34–1.95) p < 0.001) [55].

Finally, the association between frailty and quality of life (QOL) after critical illness has yet to be clarified in our population. Indeed, few studies are specific to older 75 year-olds. Several studies in healthy over 65 year-olds found that a major concern is becoming dependent [64, 65], but this is certainly subjected to individual and cultural preferences. Baseline frailty in older 65 year-olds (defined by the CFS, both used as a dichotomous variable and a continuous variable) was significantly associated with self-reported decrease in QOL (measured using the five-level EuroQol five-dimensional questionnaire (EQ-5D-5L) 6 months after critical illness [66]. In contrast, Baldwin noted that ICU survivors older than 75 or 80 years seem to accommodate to a degree of physical disability, and still report emotional and social well-being [67]. However, the data was subjected to survival, disability and proxy bias. Thus, doctors must take particular care when using frailty and estimated QOL after critical illness to justify limiting the admission of over 75 year-olds to ICUs. Indeed, a doctors’ evaluation of patient QOL may be related to their own projections as opposed to a patient’s true thoughts on the matter.

### A shift in the use of frailty scales during the Covid-19 pandemic: from the evaluation of a given patient to its adoption as a triage tool

The first wave of the Covid-19 pandemic inflicted unprecedented stress upon healthcare resources. Because of the large numbers of patients (including many older adults), presenting with rapid-onset, acute respiratory symptoms, hospital and intensive care structures were subjected to severe pressure. For this reason, healthcare professionals sought prognostic tools to help with triage, to estimate which patients might most benefit from high intensity care. Frailty assessment via the CFS had drawn the attention of the intensive care community in the late 2010s due to proven associations with mortality and loss of functional ability. Thus, the CFS seemed to be a reliable tool to help define which patients might benefit from intensive care.

Indeed, the CFS was found to be significantly associated with mortality in over 75 year-olds with Covid-19. Age and place of living, however, were not, highlighting the importance of a more complex evaluation. One multicentric prospective cohort including over 700 patients over 80 years of age found that for every CFS increase, the odds ratio for mortality was 2.2 in those not developing respiratory failure vs 1.3 (in those developing respiratory failure. Age categories and place of living (nursing home vs home-dwelling) was not associated with in-hospital mortality [68]. Covino et al., in a single-center, prospective observational cohort, found that the HR for in-hospital mortality was 6.93 in “vulnerable” (CFS 4–6) patients vs fit patients (CFS 1–3), and 12.55 in “frail” patients (CFS 7–9). Age was not a significant predictor for death.

A recent systematic review concluded that the CFS was significantly associated with mortality in Covid-19 patients. Patients with frailty (CFS 4–9) had a higher risk of mortality (OR = 3.12) and were less likely to be admitted to the ICU (OR 0.28) The authors then recommend that the CFS should be included in international Covid-19 treatment guidelines [69]. Another systematic review found that frailty in the ICU, assessed by the CFS, was significantly associated with 1 month and 3 month mortality respectively in two studies [70].

The CFS was used as a triage tool during the Covid-19 pandemic in allocating scarce health-care resources. The initial National Institute for Care and Excellence (NICE) guidelines from March 2020, which were then retracted, recommended using a cut-off of CFS ≥ 5 to limit admission to the ICU. Rockwood [30] and Hubbard [71] both highlighted the benefits and risks in using the CFS at the time for triage purposes. The use the CFS was put to during the pandemic, instrumentalized as a triage tool, was very different from previously, when it was employed to establish goals of care in a given patient. Rockwood and Hubbard warned against the extensive gap in clinical presentation and prognosis between “CFS 5” and “CFS 8” patients, all thrown together in one category of patients for whom intensive care was considered inappropriate. Thus, they strongly advised against using the CFS as a sole screening tool. Halpin cautioned against the widespread use of the CFS by teams under pressure of scarce resources. If improperly used, the notion of frailty might not apply and clinicians risk losing the confidence of people with chronic conditions or disabilities [72].

We can imagine that if one day ICU admission guidelines for older adults emerge, the CFS might be part of the triage algorithm. Consequently, it is important to be aware of the implications of using the CFS, as well as those of scales or admission algorithms in general.

## Use of scales in emergency situations for critically ill older adults

### Advantages of using scales in decision-making

Frailty is associated not only with short and long-term mortality, but with other relevant outcomes, such as being discharged home, functional mobility and health-related quality of life. For this reason, frailty could be included, among other parameters like baseline functional status, in discussions regarding escalation of care, care intensity, and goals of care [4, 73].

Scales provide physicians of different specialties with mutual, valid, language. This is especially necessary when dealing with critically ill patients. Time constraints may play an important role in the decision-making process, and various specialists are required to collaborate for patient assessment. Daily use of scales enables ongoing prospective studies (especially regarding the association between these scales and prognosis) and further scientific validation.

The CFS scale mainly requires taking a proper history of the patient to establish baseline status. Baseline status is defined as the state the patient was in 2 weeks prior to the acute episode. Geriatricians and intensivists alike using the CFS are assured that more than age and chronic diseases are taken into account. In fact, though the CFS is known as a “frailty” scale, it encompasses functional autonomy and comorbidity features. The last stages of the scale in particular allow for a finer evaluation of cognitive aspects. Thus, using this scale might be useful to have a broader perception of a patient’s health status.

### Caution is needed when using scales to aid medical decision-making

#### Nevertheless, these types of scales should be used carefully for different reasons

Firstly, there are issues about categorizing the patients via such tools. We could say that the CFS provides clinicians with a “shortcut” to CGA. It renders accessible the global evaluation process used daily in geriatrics, as opposed to a reasoning process based only, or mostly, on age. Thus, the CFS is useful in differentiating so-called “chronological age” from “biological age”. It may help diffuse evaluation and thought processes constitutional to geriatrics outside of the geriatric sphere, as such contributing to the fight against ageism. However, the use of numbers for the score might also encourage drastic categorization and thus promote stereotypes, “CFS-1” patients being the ones that are the fittest, i.e., older marathon-runners; “CFS 7–9” patients being seen as severely ill, bedridden patients, very near death. The admission of patients considered “frail” (so a CFS of 5 or more) is at risk of being dismissed on principle. For this reason, widespread instruction of the CFS must be available to clinicians using it to help decision-making. An assessment of the CFS by two different specialists when possible (for example, a geriatrician and an intensivist) is advisable, especially when the CFS is used as a triage tool among others. The identification of phenotypes with specific geriatric and acute characteristics, found to be associated with ICU mortality, may help move past an evaluation based only on the CFS [74].

Secondly, the use of the CFS during the Covid-19 pandemic in allocating scarce health-care resources, though a convenient triage tool, may have become a source of discrimination based on age (as the tool was used mainly in over 65 year-olds), as well as based on functional disability and cognitive status. It is possible that using a relatively new visual scale, depicting older adults in various states of movement and with assorted mobility aids, may have inappropriately abridged the reasoning process, especially when time was short, in teams under duress. To this end, Rockwood [30] and Hubbard [71] emphasized the need to consider the CFS a tool among others, not a discriminatory element regarding ICU admission.

Thirdly, one must take care to be precise and use the term “functional autonomy scales” as opposed to simply “autonomy”. Autonomy refers, in bioethical terms, to one’s decision-making capacity and way of deciding for oneself. We believe that the confusion and overlap between these different concepts partially foster older adults’ reduced consultation about one’s goals of care, especially when dementia is involved.

Fourthly, statistical measures do not resolve prognostic questions for a given individual, despite studies with a high level of evidence showing the influence of an ADL score of less than 3 or a CFS score of 5 or more on prognosis (both mortality and morbidity) after an acute illness. Indeed, these results might also be partially linked to self-fulfilling prophecies. This phenomenon was previously described in decision-making after severe strokes [75]. Dot-Not-Resuscitate orders and decisions to withhold/withdraw treatments were likely to be more frequent in severely ill patients with a poor National Institute of Health Stroke Scale Score (NIHSS) score. This leads to fewer interventions and a higher death rate, thus reinforcing the poor prognosis estimates made via the NIHSS score. This so-called “vicious circle” of self-fulfilling prophecies had also been previously described in the ICU [76]. Thus, being conscious of these risks is essential, whether during ICU admission processes or in decision-making in ICUs.

We need to exercise particular care when evaluating patients, taking care to use other elements in addition to scales. Scales used on their own might lead to fast, unequivocal decision making. Person-centred thinking must prevail, taking into account patient history, patient’s current and / or their surrogate’s opinion when possible, as well as their advanced directives.

Finally, social isolation and socio-economic status may explain long-term recovery after critical illness. Falvey et al. found that social isolation in older adults was significantly associated with disability and long-term mortality after critical illness [77]. They used a validated measure of social connectedness with partners, families, and friends as well as participation in valued life activities to estimate social isolation. They hypothesised that biological impairments (changes in neuroendocrine function and in systemic inflammation biomarkers), as well as challenges in accessing health care services (both medical and technical) might explain these results. Jain et al. found socio-economic disadvantage, defined by being dually eligible to Medicaid and Medicare, was associated with a 28% increase in disability after ICU hospitalization. There was a nearly tenfold risk of transitioning to probable dementia [78]. Both studies highlight the vulnerability of these populations after critical illness. It is the responsibility of healthcare providers to take these facts into account. One might argue that these facts are tricky to assess in emergency situations during the initial discussion regarding escalation of care. However, they might be taken into account once the initial critical situation is resolved, during further discussion of goals of care and when planning post-ICU care.

### The impact of ICU bed-availability and of the healthcare system

ICU bed availability has been shown to affect triage [79]. The number of ICU beds varies between countries, Iceland having the lowest proportion in Europe (at 4.8 ICU beds per 100 000 people), and the Czech Republic having the highest (45/100 000) [80]. In comparison, the USA has a stronger provision of ICU beds than most European countries (21.1 per 100 000), though France and Germany are among the exceptions. Wunsch et al., in 2011 (a time when the USA had seven times as many ICU beds as the UK), found that patients admitted to ICUs in the USA were both older and less severely ill than in the UK [81]. Wernly et al. found that European countries with a social health insurance system tended to admit a higher proportion of older patients and frail patients than tax-based healthcare systems [82]. A subgroup analysis of the VIP studies involving over 80 year-olds found that countries with a lower healthcare expenditure tended to admit patients that have a higher severity of organ dysfunction and then receive more aggressive care [83]. Thus, we must bear in mind when taking into account frailty outcomes after critical illness that patients may not have the same characteristics (both in terms of illness severity and of access to care) across countries.

### Ageism is ever-present in medicine

Ageism is widespread in medicine as well as in society [84, 85]. Ageism in medicine also results in less access to innovative research and appropriate care. Furthermore, negative ageist stereotypes have been associated with decreased will-to-live and internalized ageism among the very old, especially when their health deteriorates [86, 87].

Despite a growing population of older adults, geriatrics infrastructures vary between countries [88, 89]. In addition, there are large discrepancies in training and education in geriatric medicine [90, 91]. Interest in pursuing a career in geriatrics is low among medical students [92]. A rotation in older age medicine has been shown to influence the way medical students think about older patients [93], as well as promote the choice of geriatrics as a specialty [94].

Ageism may affect medical decision-making. What doctors themselves think is important in critical care situations. Age-related stereotypes as well as their own opinions of disability and dementia may influence their decisions [95]. Discussion about goals of care with older patients in critical situations rarely take place, even when they would be possible [96]. Clinicians themselves are poor predictors of what patients might want [97], and of their perceived quality of life [98]. Focus on advance care planning and defining goals of care is essential to facilitate patient centred care. Further research is needed, so as to improve knowledge in these areas. Meanwhile, frailty scales aspire to an evaluation of older adults that is broader than chronological age, aiming to reduce the risk of ageist decisions.

## Conclusion

In this narrative review, we explore the different scales that are used by clinicians to evaluate critically ill older adults, discuss which tool is best to evaluate frailty in these situations, as well as the biases, particularly the risk of caricature or patient discrimination based solely on age or on functional autonomy.

Scales provide emergency physicians, geriatricians and intensivists with a common language and validated communication tools, moving beyond a patient’s chronological age. The CFS, a scale which differs from the FP, encompasses functional autonomy and comorbidity features. It is easy to use and reliable. It has been shown to be associated with short and long-term mortality, as well as with disability and discharge to home after a critical illness. Without these validated tools, older adults may not benefit from appropriate care in acute situations. However, the improper use of scales may lead to hastened decision-making, especially when there are strains on healthcare resources or time-constraints. Frailty and functional autonomy scales, used on their own risk, risk “reducing the patient to a number”, thus taking away the focus from the patient as an individual. Physicians must continue to present full context and take into account prior goals of care discussions and advanced directives, as well as a full history of the patient, both medical and in terms of in depth personality evaluation, thus encouraging an integrative approach to critical care decision-making [99]. Although more time consuming, these measures are necessary to prevent the risk of oversimplifying complex situations and allocating treatments to a given patient using only scales. Thus, doctors may hope to sustain a patient-centred approach and ethical decision-making.

## Data Availability

All data and materials may be requested to the corresponding author: Dr Laura Moïsi.

## Abbreviations

ADL: Activities of Daily Living
ED: Emergency department
IADL: Instrumental Activities of Daily Living
CCI: Charlson Comorbidity Index
CFS: Clinical Frailty Scale
CGA: Comprehensive geriatric assessment
CHSA: Canadian Health Studies in Aging
CIRS-G: Cumulative Illness Rating Scale for Geriatrics
FI: Frailty Index
FP: Frailty phenotype
HFRS: Hospital frailty risk score
ICU: Intensive care unit
NICE: National Institute for Care and Excellence
NIHSS: National Institute of Health Stroke Scale Score
QOL: Quality of life
PS: Performance status
VIP: Very elderly intensive care patient
